# A successful stacking of multiple biotic stress resistance in rice facilitated by genes and precision breeding tools

**DOI:** 10.1186/s40659-026-00710-0

**Published:** 2026-08-05

**Authors:** Muthukumarasamy Sriram, Kailappan Amudha, Swaminathan Manonmani, Chellapan Gopalakrishnan, Venugopal Sheela, John Jesu Bonipas Antony, Selvaraj Vignesh, Ramalingam Suresh

**Affiliations:** 1 Department of Genetics and Plant Breeding, CPBG, Tamil Nadu Agricultural University, Coimbatore, 641003 India; 2 Agricultural Research Station, Bhavanisagar, 638451 India; 3 Department of Rice, CPBG, Tamil Nadu Agricultural University, Coimbatore, 641003 India

**Keywords:** Multi-genic resistance, Blast, Bacterial leaf blight, Brown plant hopper, Gene pyramiding, Marker-assisted breeding

## Abstract

**Background:**

Genetic improvement of rice for biotic stress is a major and continuous breeding objective owing to changing pest and disease scenarios. In response to climate change, an outbreak of new pathotypes and biotypes results in huge yield loss which directly impacts the economic stability and food security. The development of varieties with multiple resistance genes for a particular disease is the most effective approach to the combating mechanism against evolving pathogens and insects. Multiple resistance genes against blast (*Pi54*), bacterial leaf blight (*xa5*, *xa13*, *Xa21*), and brown plant hopper (*Bph17*, *Bph3*, *bph2*) were pyramided through marker-assisted forward breeding by attempting multiple crosses involving six parental lines. The F_4_ lines were screened and the resistant lines were further reconfirmed at the F_5_ stage through precise phenotyping and also by genotyping with trait-specific markers. Finally, the selected lines were evaluated for the agronomic performance.

**Results:**

Four lines were selected from the population which carrying seven resistance genes against BL, BB, BPH with superior agronomic performance. Another line, x21302-239, harboring all the resistant genes and showing a resistant response in screening experiments for all three stresses, with marginal agronomic performance (single-plant yield: 33 g), can be utilized as a desirable donor to develop elite rice cultivars with multiple biotic stress resistance.

**Conclusions:**

Homozygous genetic background is more favorable for epidemic outbreak in a short period of time in comparison with the population of multiple genetic background. Since this complex genetic background disturbs the infectious cycle of the pathogen. So that a variety or a hybrid which developed from multiple parental lines are notable for their durable resistance than the monogenic resistance variety.

**Supplementary Information:**

The online version contains supplementary material available at 10.1186/s40659-026-00710-0.

## Background

Rice (*Oryza sativa* L.) is a predominant commodity in the global food market, which ensures the food security, and economic stability of the rice-growing nations. It supports more than 20% of the world’s dietary energy and acts as a stable food crop for more than 3.5 billion people [18, 42, 61]. The rising global population is demanding a continuous increase in rice production, and it is estimated that an additional 116 million tons of rice is required by 2050, beyond current production levels, to meet the food demands. However, globally rice production is inevitably affected by many constraints such as limited arable area, water scarcity, emergence of new pathogens and insects, and credibly adverse effects of climatic change [55], [61]. The rice production is consistently affected by various biotic and abiotic stress. To date, more than 70 diseases and 800 insect species have been reported to affect the rice in field and storage conditions [2, 59]. Thus, breeding efforts were made to develop multiple biotic stress-resistant cultivars through marker-assisted introgression of genes governing resistance to key pests and diseases of rice.

Rice blast (BL) is a serious disease caused by the fungus, *Magnaporthe oryzae* which infects the leaves, neck, and nodes of the crop. Severe infection of the disease causes yield loss of more than 90% [2]. Initially, it was identified in California in 1996 [21]. The blast fungal pathogen sustains in the plant debris such as diseased straw, seed, ratoons, and volunteer plants in the form of mycelium and conidia. The initial symptom of the blast disease is the formation of lesions with grey to deep brown colour in size of 1–2 mm [48]. Many resistant genes/QTLs have been identified from various landraces, and wild relatives through the phenotypic screening process and molecular approaches for various pests and diseases. So far, over 100 resistance genes and QTLs for the blast disease, among them 37 genes were cloned [43]. Two dominant resistant genes, *Pi54* and *Pi1* were reported to confer resistance against major virulent races of the pathogen in India and were widely used in most of the research programmes [27].

Bacterial leaf blight (BB), caused by *Xanthomonas oryzae* pv. *oryzae* is one of the serious diseases with a huge yield loss of 80% under severe infection. It was first reported in Japan in 1884 and is now most prevalent in rice-growing countries [44]. *Xanthomonas oryzae* pv. *oryzae* infection initially induces streaks on leaves that spread from the tips and margins. These streaks become larger and eventually release a milky fluid that dries yellow. Late stages of infections are characterized by greyish-white lesions on the leaves, eventually resulting in the leaves drying out and dying [47]. The yield loss incurred by the BB pathogen is due to the reduced photosynthesis rate, blockage of water supply, and increased transpiration rate [1, 12]. For improving the host-plant resistance against the *Xoo* pathogen, till date, more than 50 resistance genes have been identified from diversified sources. Out of these genes, *Xa4*, *xa5*, *Xa7*, *xa13*, *Xa21*, *Xa33*, and *Xa38* genes were widely harbored in the BB resistance breeding programme [8, 43]. The *Xa21* resistant confers a broad spectrum of resistance over a wide range of *Xoo* isolates. Distinct from other resistant genes, *xa5* encodes for a gamma transcription factor-like protein, and in combination with other dominant resistant genes it provides durable resistance against the *Xoo* pathogen. Another recessive resistant gene, *xa13* resulted from the mutation reaction of the promoter region of the dominant allele of *Xa13* [6, 22, 25].

The Brown Plant Hopper (BPH) is the most destructive insect pest that threatens rice production globally. There are four biotypes of BPH populations documented, out of which, the most hazardous biotype particularly in the Indian subcontinent, is biotype 4 [40]. The BPH insect, pierce and sucks the plant sap from phloem tissue. with a modified mouthpart called a stylet [52]. As it consumes phloem sap, eventually causes plant death and substantial yield losses [19]. The damage is caused either by loss of sap from phloem or by the injection of toxic saliva. This symptom is known as “hopper burn” [52]. Due to the saliva deposition, the formation of a fungal colony called sooty mold has a negative impact on plants. It accounts for about 20–80% of yield loss and an overall economic loss of around 252 core rupees in Asia annually [36]. About 40 BPH resistance genes were reported from various landraces and wild relatives, in which most of them are present in clusters on chromosomes 3, 4, 6, and 12. Particularly, 17 resistance genes, viz., *Bph14*, *Bph17, Bph15*, *Bph26/bph2*, *bph29*, *bph7/Bph9/Bph10/Bph21*, *Bph18/Bph1*, *Bph32*, *Bph6*, *Bph30/Bph40*, and *Bph37* were isolated and characterized through map-based cloning method [29, 32, 33].

The development of monogenic genic resistance against any specific disease or insect is vulnerable to the rapidly evolving adaptation mechanism of the insect/pathogen. Which sequentially results in the breakdown of resistance in the host plant [50]. Therefore, the pyramiding of multiple resistance genes against the specific disease or insect improves the durability of resistance and also the level of resistance [10]. In addition, combining or pyramiding genes conferring resistance to multiple biotic stresses holds a promising approach to tackle multiple pests and diseases with a single variety. This can be achieved by integrating molecular approaches with classical breeding, which helps in the precise and simultaneous introgression of two or more resistance genes in different genetic backgrounds. Similarly, through pyramiding approach coupled with marker-assisted selection (MAS), three blast resistance genes such as *Pi54*, *Pi1*, and *Pita* were introduced into IET 25480 and released as the *Pusa Samba* variety [2]. An elite cultivar Krishna Hamsa was improved with resistance to blast, BB, BPH, and tolerance to drought stress through multiparent crosses along with marker-assisted introgression of resistance genes/QTLs [3].

 Keep in the view of aforesaid, the aims of the present study are, (1) development of multiple resistant populations through gene pyramiding approach, (2) identification of multi-resistant lines through morphological screening, and molecular validation using gene specific markers, and (3) evaluation of identified resistant lines for the agronomic performances.

## Materials and methods

### Crossing of parents and development of the population:

**Fig. 1:**
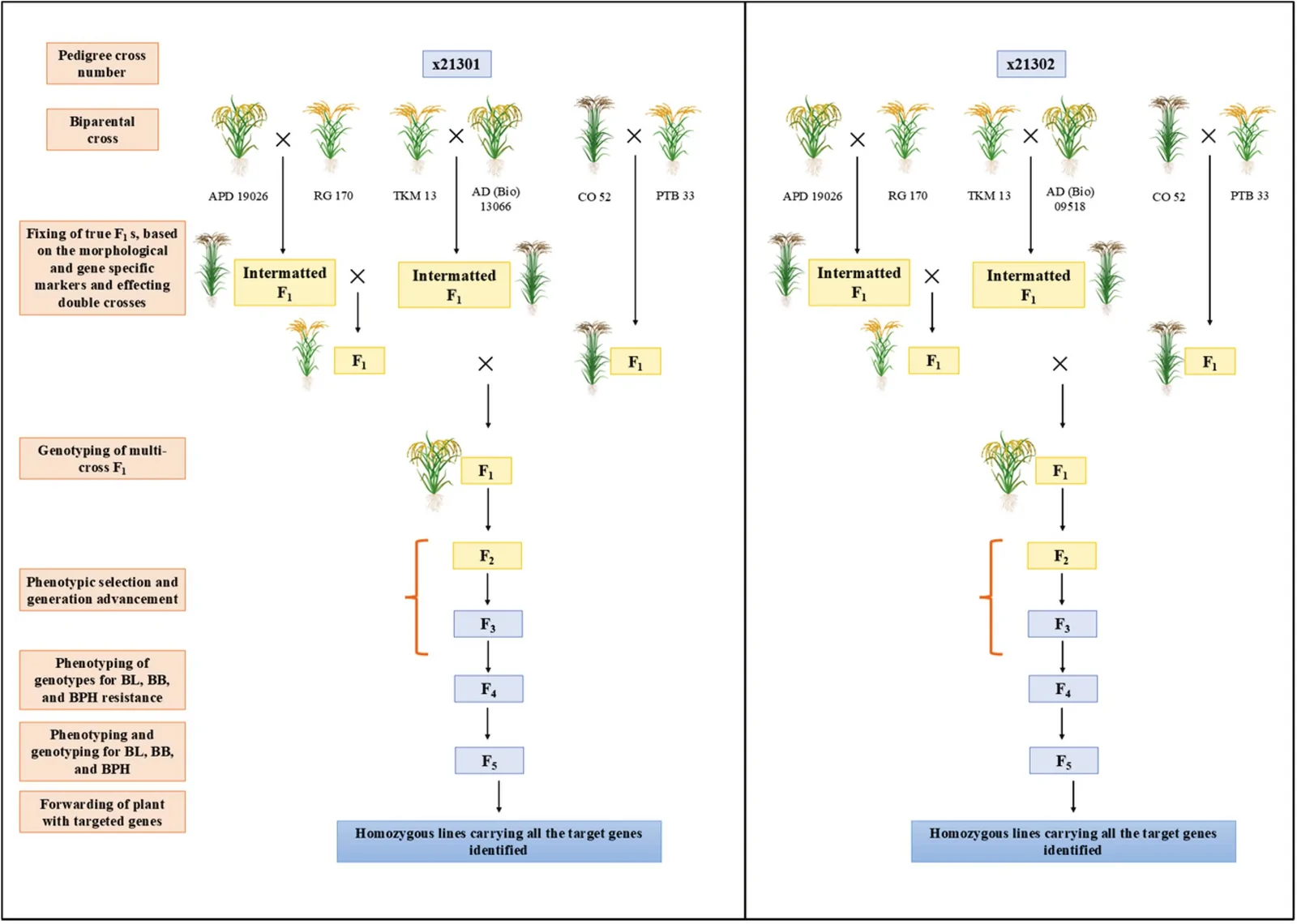
Schematic representation of the breeding schemes for x21301 and x21302, delineating the systematic process from parental crosses through generation advancement to the identification of homozygous lines with pyramided BL, BB, and BPH resistance genes

The development of multiple biotic resistance lines specifically for the blast (BL), bacterial leaf blight (BB) diseases, and brown plant hopper (BPH) was developed through the gene pyramiding approach. Initially, two different crosses were made with seven parents viz., APD 19026 (Improved Samba Mahsuri) for blast resistance genes, AD (Bio) 09518 and AD (Bio) 13,066 for bacterial leaf blight resistant genes, and PTB 33 for brown plant hopper. RG170 was used as a donor parent for false smut resistance. However, it was not studied extensively due to non-congenial weather for the disease occurrence and also due to non-availability of artificial screening facility for this disease. The characteristics of parental lines were listed in the supplementary Table 1 (Table S1). The F_1_s of the biparental crosses between ISM x RG 170, and TKM x AD (Bio) 13,066 were crossed and the first intermated F_1_ was developed. For the second cross, the intermated F_1_s were developed by crossing two F_1_s, such as ISM x RG 170, and TKM x AD (Bio) 09518. The intermated F_1_s of both crosses were again crossed with the F_1_s of the cross CO 52 × PTB 33 (Fig. 1). Both the multiple crosses were designated with the cross numbers x21301 and x21302. A multiple-cross true F_1_ population derived from these crosses was selected with the presence of targeted genes through molecular analysis. Eight target-positive plants from cross x21301 and ten plants from cross x21302 were identified and forwarded to the F_2_ generation. Together from both the F_2_ populations, about 270 phenotypically superior single plants out of 2200 plants were selected without genotyping and advanced to F_3_ and later to F_4_ generations, which formed the basic breeding material for screening against the major biotic stresses of rice viz., blast, bacterial leaf blight, false smut and brown plant hopper.

### Screening for blast resistance in the uniform blast nursery (UBN)

**Fig. 2:**
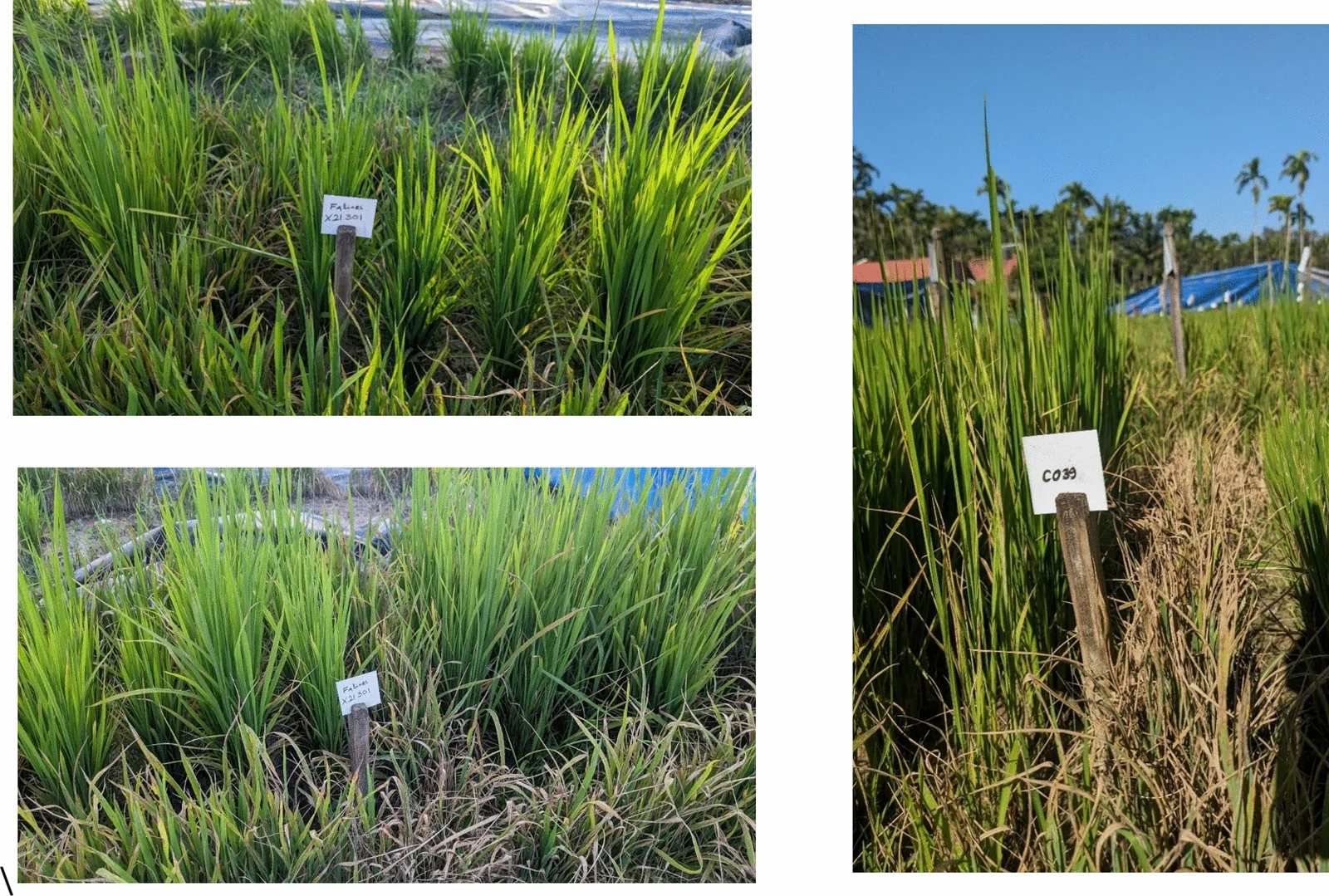
Blast resistance screening was conducted at the hotspot location of HREC, Gudalur under natural field conditions. The experimental layout included a susceptible check, CO 39, planted in a 4:1 ratio, comprising four rows of each test entry alternated with one row of CO 39

The F_4_ population (270 progeny rows) was screened against blast disease in the natural hotspot condition, Hybrid Rice Evaluation Centre (HREC), Gudalur following the uniform blast nursery (UBN) screening method [7]. During *Rabi* 2023 all the 270 homozygous F_4_ lines and their parents along with susceptible check CO 39 were sown in a blast nursery (Fig. 2). The susceptible check CO 39 was raised in border rows and also after every five rows of the test genotypes as a spreader row to enhance and ensure the infestation. Disease scoring was taken at 30 and 45 days after sowing based on the standard evaluation system, [23].

The highly resistant, resistant, and moderately resistant F_4_ lines were advanced to F_5_ and were screened again during the wet season 2024 to reconfirm the resistance of the resistant F_5_ lines identified through preliminary screening to confirm the resistance reaction shown by them by conducting the similar experiment at the same location (HREC, Gudalur).

### Screening for bacterial leaf blight resistance by clipping method

A total of 51 blast-resistant and moderately resistant lines identified with the scores 1 and 3 at HREC, Gudalur were tagged and shuttled to Paddy Breeding Station, Coimbatore, and planted in the main field for artificial screening against BB. Artificial inoculation was carried out by clipping the leaves with BB spore-smeared scissors at the profuse tillering stage. The BB pathogen was collected from Coimbatore and Gudalur regions at a concentration of 1 × 10^8^ CFU/ml. The inoculated leaf samples were scored 1 week after inoculation based on the standard evaluation system, IRRI [23]. At maturity, seeds were harvested from all 51 F_4_ lines and utilized for screening against BPH and confirmatory screening for blast.

### Screening for brown plant hopper resistance

The 51 blast-resistant lines at F_5_ generation were screened for BPH, at the Entomology Glass House of Paddy Breeding Station during summer and wet seasons, 2024. Initial 51 genotypes were screened at the seedling stage in standard seed box test (SSBT), and then the resistant lines identified were screened in the modified standard seed box test (MSSBT) to confirm the resistance reaction. Damage score of the test entries was done when 90 percent of the susceptible check-TN1 exhibited wilting symptoms based on the standard evaluation system of IRRI [23].

### Validation of resistance genes by molecular markers

The genomic DNA was extracted following the CTAB protocol from 51 F_5_ lines and seven parents. The polymerase chain reaction (PCR) protocol for the marker-aided selection (MAS) of the targeted genes/QTLs was followed [26]. Validation of resistance genes was carried out with the gene-specific markers for each resistant gene. The list of resistant genes and molecular markers used in the validation approach are listed in Table 1.

**Table 1:** Molecular markers linked with the *Pi54*, *xa21*, *xa5*, *Xa13*, *Bph1*, *bph2*, *Bph13*, and *Bph33(t)* resistance genes

S. No	Gene/QTL	Primer	Chromosome number	Forward sequence	Reverse sequence	Annealing temperature
1	*Pi54*	*Pi54*-MAS	11	CAATCTCCAAAGTTTTCAGG	GCTTCAATCACTGCTAGACC	55
2	*Xa21*	pta248-Xa21	11	AGACGCGGAAGGGTGGTTCCCGGA	AGACGCGGTAATCGAAAGATGAAA	59
3	*xa5*	RM153	5	GCCTCGAGCATCATCATCAG	ATCAACCTGCACTTGCCTGG	54
4	*xa13*	xa13-prom	5	TCTTGCCCGTCACTGCAGATATCC	GCAGCCCTAATGCTACAATTCTTC	57
5	*Bph3*	RM273	4	GAAGGCCGTCGTGAAGTTACC	GTTTCCTACCTGATCGCGAC	55
6	*bph2*	RM463	12	TTCCCCTCCTTTTATGGTGC	TGTTCTCCTCAGTCACTGCG	53
7	*Bph1*	RM512	1	CTGCCTTTCTTACCCCCTTC	AACCCCTCGCTGGATTCTAG	54
8	*Bph33(t)*	RM212	1	CCACTTTCAGCTACTACCAG	CACCCATTTGTCTCTCATTATG	51

### Biochemical confirmation of blast resistance

A total of 10 highly resistant and resistant lines viz., x 21301-38, x21301-52, x21301-147, x21301-150, x21302-151, x21302-239, x21302-240, x21302-241, x21302-252 and x21302-270 along with the donor parent APD 19026 and susceptible check CO 39 were evaluated for the activity of phenylalanine ammonia-lyase, peroxidase and ascorbate peroxidase enzymes as per the methods described by Sadasivam & Manickam [46], and Nakona & Asada [37] respectively. The samples for the enzymatic analysis were taken at 0, 3, 9, 12, and 15-day intervals after inoculation of the pathogen.

### Confirmation of brown plant hopper resistance by honeydew test

The resistant lines identified based on MSSBT were subjected to a honeydew excretion test, which determines the insect’s feeding activity. This method involves the collection of hopper-excreted honeydew on the filter paper and measuring the area of honeydew deposition on the filter paper. The filter papers were treated with 0.01% ninhydrin solution and dried till the violet or purple colour appeared due to the amino acid content in the honeydew. The measurement of the area covered with violet or purple colour in the filter paper was done using the Digimizer software.

### Evaluation of agronomic performance and yield analysis

The selected 51F_5_ lines were evaluated for the agronomic performance in Augmented Block Design II (ABD II) at the Paddy Breeding Station, Coimbatore during the *Summer*, 2024. The biometrical observations for the growth and yield-related traits such as, days to 50% flowering (DFF), plant height (PH), panicle length (PL), number of productive tillers (NPT), single plant yield (SPY), and thousand-grain weight (TGW) were recorded in both F_4_ and F_5_ generations.

### Statistical analysis

The data obtained from the experiments were statistically analyzed using analysis for variance (ANOVA) of the ABD II. The genetic variability parameters of each trait, the correlation between the traits, and the principal components for the yield and related traits were analyzed in the F_4_ and F_5_ generations. The effect sizes were estimated using Eta squared (η^2^), calculated as the proportion of total variance attributable to treatment effects. The observed resistance scores for each stress were statistically validated using Duncan’s test. The statistical analysis was conducted in the R studio with the packages augmentedRCBD, metan, corrplot, MASS, ggplot2, factoextra, and agricolae.

## Results

### Identification of BL, BB, and BPH-resistant lines in F_4_ population

Out of 270 F_4_ lines screened in uniform blast screening nursery under hot spot location, three lines viz., x21302-151, x21302-240, and x21302-241 were identified as highly resistant with a score of 1.0 and without any lesions, 8 lines (x21301-2, x21301-38, x21301-131, x21302-166, x21302-193, x21302-239, x21302-246 and x21302-268) were found to be resistant with minute brownish non-sporulating spots of pinhead size and the disease reaction score of 2.0. Another 67 lines with a score of 3.0 fall under the moderately resistant category.

Screening against BB through artificial inoculation of selected 51 lines showed, 10 genotypes viz., x21301-32, x21301-52, x21301-74, x21301-98, x21301-100, x21301-132, x21302-152, x21302-161, x21302-240 and x21302-241 were highly resistant with a score of 1.0 and percent leaf area infection of 1–5%. Another 16 lines with a score of 3.0 were found to be resistant.

The same set of 51 lines were screened against BPH through a standard seed box test (SSBT), which resulted in the identification of 6 resistant lines viz*.,* x21301-52, x21301-147, x21301-150, x21302-239, x21302-252, and x21302-270 with an average score of 3.0 and the symptom of wilted lower leaf with two green upper leaves. Two F_4_ lines, x21301-26 and x21301-84 classified under moderately resistant category with a symptom of wilted two lower leaves with one green upper leaf were identified. In the confirmation study, one out of 8 lines (x21302-239) was consistent for its resistance reaction, and the remaining seven lines, x21301-52, x21301-147, x21301-150, x21302-252, x21302-270, showed moderate resistance.

### Selection of F_4_ lines conferring multiple biotic stress resistance

Phenotypic screening of F_4_ lines for the BL, BB, and BPH leads to the identification of a line, x21302-239 having combined resistance to all three biotic stresses. Two lines viz., x21302-240 and x21302-241 were found to have resistance to BL and BB. One line—x21301-150 expressed resistance to BL and moderate resistance to BPH whereas the line x21301-52, showed moderate resistance to BL and BPH and it was highly resistant to BB. Moderate resistance to BL and resistance to BB was noticed in eight lines viz., x21301-32, x21301-52, x21301-74, x21301-98, x21301-100, x21301-132, x21302-152, and x21302-161 on contrary two other lines (x21301-38 and x21302-239) were identified with resistance to BL and moderate resistance to BB. Moderate resistance for both BL and BB was observed in eight lines (Table S2).

### Confirmation of blast, BB, and BPH-resistant lines in the F_5_ population

All 51 blast-resistant F_4_ lines were advanced to F_5_ generation and screened again at the hotspot location to identify the consistent lines. Confirmatory screening validates the highly resistance reaction against blast in four lines viz., x21301-38, x21302-151, x21302-240, and x21302-241 and eleven lines (x21301-2, x21301-26, x21301-41, x21301-52, x21301-53, x21301-99, x21301-131, x21301-132, x21301-150, x21302-239 and x21302-246) were found to be resistant. The remaining 36 lines fall under the moderate resistance category.

Similarly, validation of 51 F_5_ lines against BB leads to identification of 10 lines (x21301-32, x21301-52, x21301-74, x21301-98, x21301-100, x21301-132, x21302-152, x21302-161, x21302-240 and x21302-241) having high level of resistance and 12 lines (x21301-33, x21301-59, x21301-64, x21301-73, x21301-98, x21301-107, x21301-146, x21302-239, x21302-243, x21302-251, x21302-252, and x21302-270) as resistant ones.

All the 51 F_5_ lines were initially screened in SSBT, in which 11 lines (x21301-52, x21301-53, x21301-112, x21301-147, x21301-150, x21302-239, x21302-243, x21302-246, x21302-252, x21302-264 and x21302-270) with a score of 2.0 were found to be resistant and four lines (x21301-41, x21301-51, x21301-26, x21302-250) registered the score 5.0 falls under moderately resistant against BPH. Combining these 15 resistant and moderately resistant lines confirmatory MSST screening was done. Which resulted in, the identification of one resistant (x21302-239) and five (x21301-52, x21301-147, x21301-150, x21302-252, and x21302-270) moderately resistant lines to BPH.

### Identification of F_5_ lines showing multiple resistance based on confirmatory experiments

**Fig. 3:**
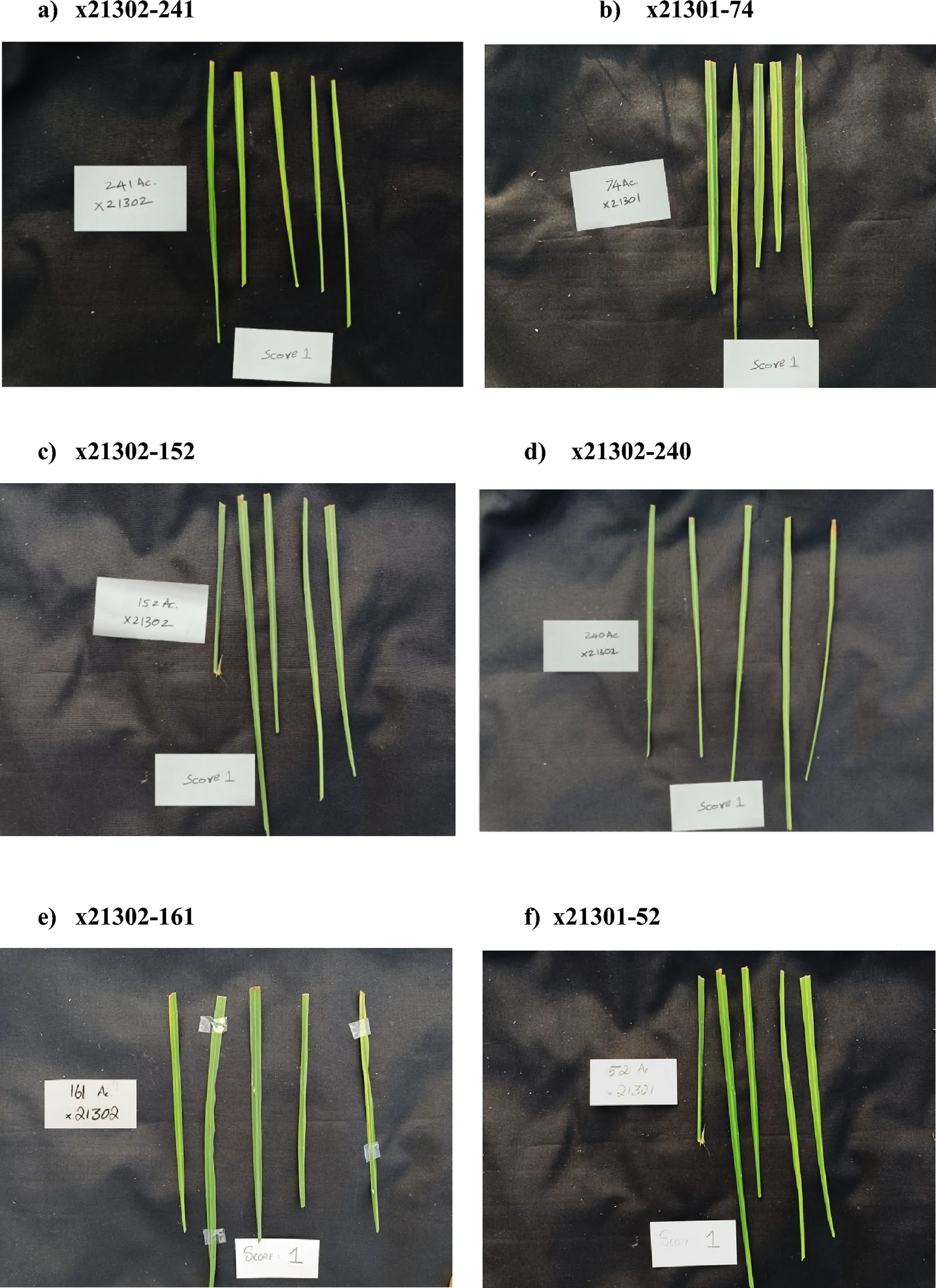
Highly resistant lines selected through artificial screening (clipping method) for bacterial leaf blight disease

One out of 51 lines showed resistance reaction to BL, BB, and BBH, and two lines viz., x21301-52 and x21301-150 showed resistance against BL and BPH along with high resistance to BB. A high level of resistance to BB, resistance to BPH, and moderate resistance to BL was noticed in x21301-147. For both blast and BB. The lines such as x21301-38, x21302-151, x21302-240, and x21302-241 showed highly resistant reactions, and another six lines (x21301-26, x21301-41, x21301-52, x21301-99, x21301-132, and x21301-150) were found to be resistant to BL and highly resistant to BB (Fig. 3). A moderate degree of resistance to blast and a high level of resistance to BB was observed in 12 lines (x21301-32, x21301-44, x21301-47, x21301-51, x21301-74, x21301-84, x21301-100, x21301-116, x21301-147, x21302-152, x21302-161, and x21302-268). A total of 11 lines viz., x21301-33, x21301-59, x21301-64, x21301-73, x21301-98, x21301-107, x21301-146, x21302-243, x21302-251, x21302-252, and x21302-270 recorded moderate resistance to blast and resistance to BB (Table S3).

### Lines conferring resistance against multiple stresses in both F_4_ and F_5_ generations

One line x21301-52 was found to be promising with highly resistant and resistant reactions to BL and BB, and BPH respectively. Likewise, x21302-239 was also consistent in its resistance reaction against BL, BB, and BPH over generations. Seven lines such as x21301-32, x21301-74, x21301-100, x21301-132, x21302-152, and x21302-161, were found to be resistant and highly resistant to BL and BB respectively. Two lines, x21302-240 and x21302-241 recorded a high level of resistance to B and BB. Moderate resistance to blast and resistance to BB was registered in the lines such as x21301-33, x21301-73, x21301-107, and x21302-243 whereas, the line x21301-38 showed highly resistance reaction for both BL and BB.

### Biochemical confirmation of blast resistance

#### Activity of phenylalanine ammonia-lyase (PAL) enzyme

**Fig. 4:**
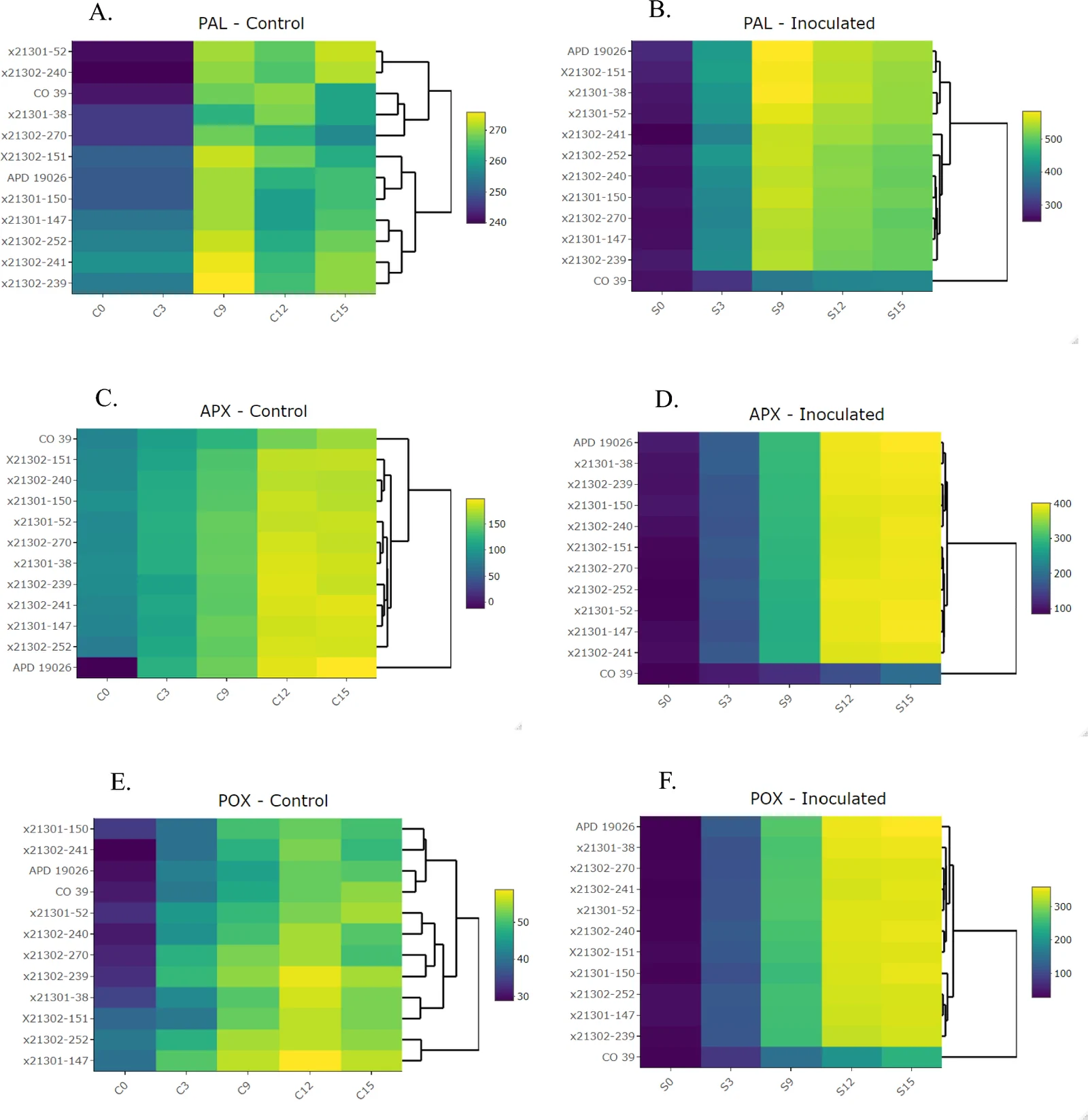
Expression of plant defense enzymes, viz., phenylalanine ammonium lyase (PAL), ascorbate peroxidase (APX), and peroxidase (POX) during resistance against the blast pathogen. (**A**, **C**, and **E**—PAL, APX, and POX expression at control, **B**, **D**, and **F** – PAL, APX, and POX expression under stress condition)

Test entries, x21301-38 (533), x21301-52 (533), and x21302-151 (535) were recorded with increased PAL activity equal to the resistant check, APD 19026 at 15 DAI. These lines have recorded with increase of 1.960, 1.99, and 1.903 folds under pathogen-infected conditions. Significantly highest PAL enzymatic activity was recorded in x21302-241 (2.071 folds) followed by x21301-52 (1.989 folds), x21301-38 (1.960 folds), and x2102-240 (1.950 folds) (Fig. 4).

#### Activity of ascorbate peroxidase (APX) enzyme

Under controlled conditions, the APX activity was recorded in a range between 199 and 168 η Moles at 15th day. While at the pathogen-infected condition, it was increased by 2.272 folds in the susceptible check and 3.688 folds in the resistant check. The ten test entries were recorded with an increased APX activity by more than 3.7 folds with a maximum of 4.528 folds in x21302-252 followed by x21301-52 (4.522 folds), x21302-270 (4.4 folds), x21302-151 (4.271 folds) and x21301-147 (4.2 folds) under the pathogen inoculated condition (Fig. 4).

#### Activity of peroxidase (POX) enzyme

The POX enzyme activity was recorded as the increase of POX activity ranged from 8.095 to 11.533 folds increase among the test entries under infected conditions. After the infection of the pathogen the test entry, x21302-241 (339) recorded with the highest increase of 11.533 folds which is similar to the resistant check, APD 19026 (11.612 folds), followed by x21301-38, x21301-52, and x21302-270 with an increase of 11.354, 10.781, and 10.424 folds respectively (Fig. 4).

#### BPH resistance confirmation through the honeydew test

**Fig. 5:**
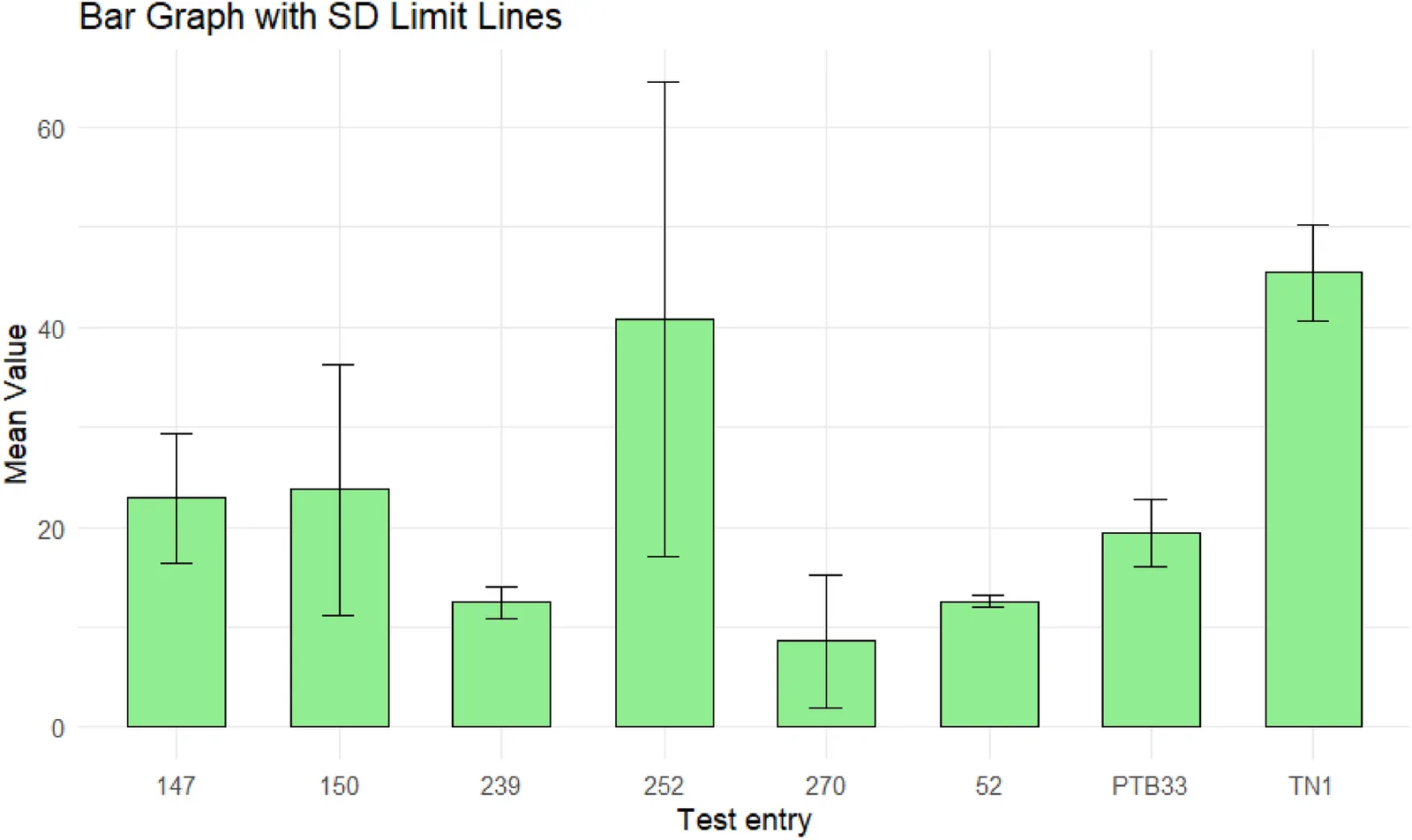
Mean value is the honeydew excretion area by the BPH insect during feeding. Test entry x21302-270 shows the minimal feeding activity, while the susceptible check (TN1) was recorded with higher feeding activity

**Fig. 6:**
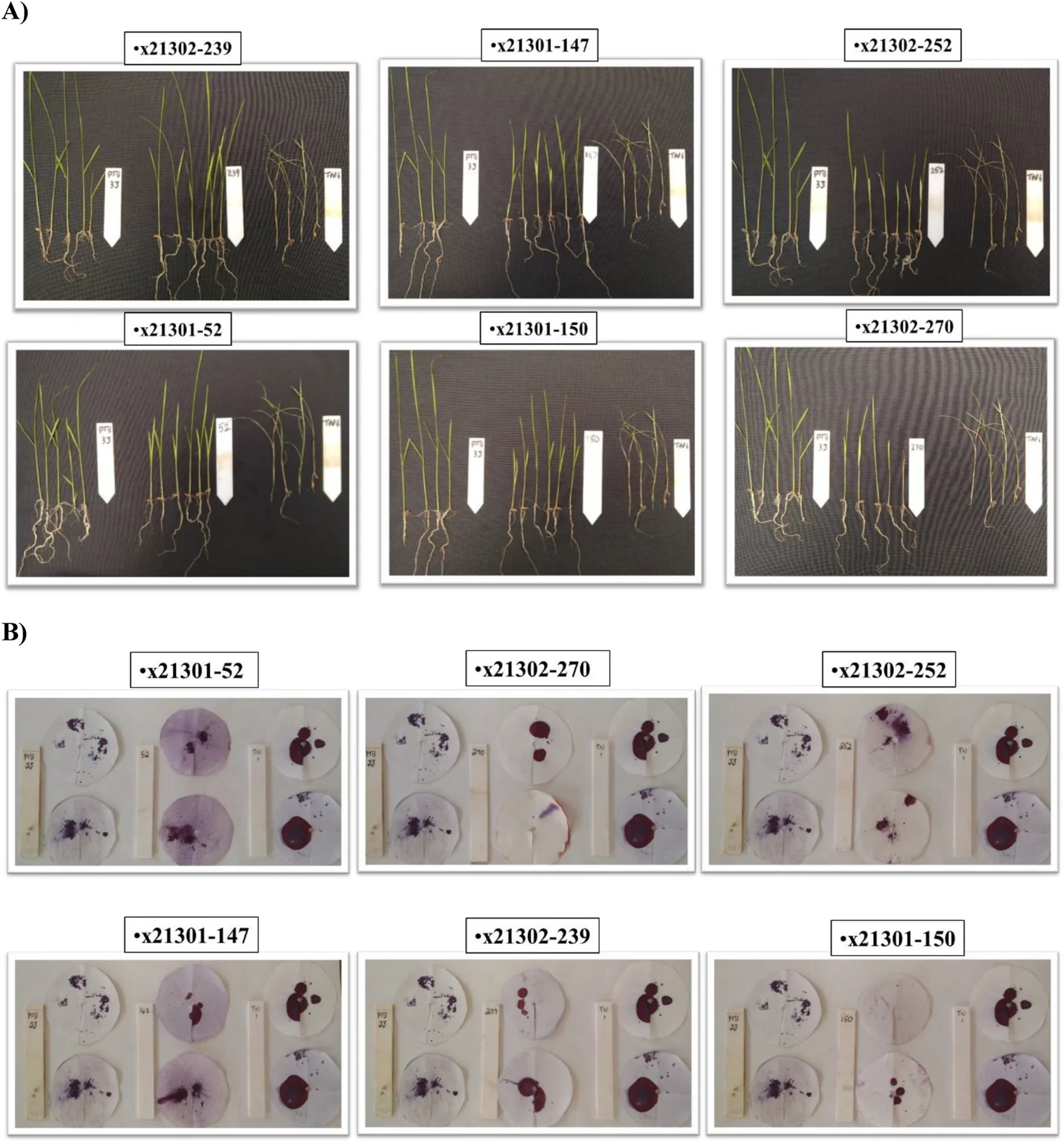
**A** Lines selected by modified standard seed-box test **B** Selected lines were subjected for estimation of Honeydew excretion by ninhydrin treatment—the purple dark areas are exudates from BPH insects after feeding. Larger purple area represents the high feeding activity of insects

The honeydew test was performed for the six resistant and moderately resistant lines viz., x21301-52, x21301-147, x21301-150, x21302-239, x21302-252, and x21302-270, identified through SSBT and MSBT screenings. All six lines along with the resistant (PTB 33) and susceptible (TN 1) were evaluated in two replications (Figs. 5 and 6). The honeydew area after a day of feeding period in PTB 33 and TN 1 was recorded with 19.441 cm^2^ and 45.449 cm^2^ respectively whereas, in the test entries, the lowest area of honeydew deposition was noticed in the lines x21302-270, x21302-239, and x21301-52. The other lines such as 21301-150, x21301-147, and x21302-252 registered more than 20 cm^2^, which is slightly higher than the resistant check value.

#### Molecular validation of resistant genes against BL, BB, BPH, and FS:

The molecular detection of the targeted resistant genes for BL, BB, and BPH by SSR markers are listed in the Table 2. The marker analysis identified four lines (x21301-1, x21301-2, x21301-47, and x21301-59) with 7 resistant genes in different combinations for BL (*Pi54*), BB (*xa5*, *xa13*, *Xa21*), and BPH (*bph1*, *Bph2*, *Bph17*) (Supplementary Fig. 1). The line x21301-2 had an extra resistant gene, *Xa21* in heterozygous condition. These four lines with 7 resistant genes were selected and forwarded for testing in yield trials. The lines, x21301-20 (*Pi54*, *xa5*, *xa13*, *Bph3*, *Bph17*), x21301-64 (*Pi54*, *Xa21*, *xa13*, *Bph3*, *Bph2*), x21301-73 (*Pi54*, *xa5*, *xa13*, *Xa21*, *Bph3*), x21302-164 (*Pi54*, *xa5*, *xa13*, *Xa21*, *bph1*, *Bph2*), x21302-239 (*Pi54*, *xa5*, *xa13*, *bph1*, *Bph2*, *Bph17*), and x21302-251 (*Pi54*, *xa5*, *xa13*, *Xa21*, *Bph3*) were identified with a combination of 6 resistant genes in unique combinations. Based on genotyping the above ten lines were identified with genes / QTLs conferring resistance to BL, BB, and BPH. Out of 51 lines, 15 and 13 lines were identified with a combination of 5 and 4 resistant genes respectively.

**Table 2:** Validating the presence of targeted resistance genes in the F_5_ lines using the gene-specific SSR molecular markers

Lines	*Pi54*	*Xa21*	*xa5*	*xa13*	*Bph3*	*Bph1*	*bph2*	*Bph17*
	Pi54-MAS	pta248-Xa21	RM153	xa13-prom	RM273	RM512	RM463	RM212
x21301-1	R	R	R	S	R	S	R	R
x21301-2	R	H	R	R	R	S	R	R
x21301-5	R	H	R	S	R	S	R	S
x21301-20	R	H	R	R	R	S	S	R
x21301-26	R	H	S	R	R	S	R	S
x21301-32	R	H	S	H	R	S	R	S
x21301-33	R	H	S	R	S	R	S	S
x21301-38	R	H	S	R	S	R	S	S
x21301-41	R	H	S	R	S	R	S	S
x21301-44	R	H	R	S	R	R	S	S
x21301-47	R	R	R	R	R	R	S	S
x21301-51	R	H	R	R	S	R	S	S
x21301-52	R	H	S	S	S	R	S	S
x21301-53	R	H	S	R	S	R	S	R
x21301-57	R	R	S	S	S	S	R	R
x21301-59	R	R	S	R	R	S	R	R
x21301-64	R	R	S	R	R	S	R	S
x21301-73	R	R	R	R	R	S	S	S
x21301-74	R	H	S	R	R	S	S	S
x21301-83	R	R	S	R	S	S	S	S
x21301-84	R	H	R	S	S	S	R	S
x21301-98	R	H	S	R	S	S	S	S
x21301-99	R	R	S	S	S	S	R	R
x21301-100	R	H	R	S	S	S	R	R
x21301-107	R	H	R	S	S	S	R	R
x21301-112	R	R	R	S	S	S	R	S
x21301-116	R	R	S	R	S	S	R	S
X21301-131	R	H	S	R	S	S	R	S
X21301-132	R	H	R	H	S	R	R	S
X21301-133	R	H	S	R	S	S	S	S
x21301-146	R	H	S	S	S	R	R	S
x21301-147	R	H	S	S	S	R	R	R
x21301-150	R	H	R	S	S	R	R	S
x21302-151	R	H	R	R	S	R	R	S
x21302-152	R	H	R	H	S	R	R	S
x21302-153	R	H	R	R	S	S	R	R
x21302-161	R	H	R	S	S	R	R	S
x21302-164	R	R	R	R	S	R	R	S
x21302-239	R	H	R	R	S	R	R	R
x21302-240	R	R	R	S	S	S	S	R
x21302-241	R	R	R	R	S	S	S	R
x21302-243	R	H	S	R	S	S	S	R
x21302-246	R	R	R	R	R	S	S	S
x21302-250	R	R	S	S	R	S	S	S
x21302-251	R	R	R	R	R	S	S	S
x21302-252	R	R	R	S	R	S	S	S
x21302-254	R	H	S	S	R	S	S	S
x21302-264	R	R	S	R	R	S	S	S
x21302-266	R	H	S	S	R	S	S	S
x21302-268	R	H	S	S	R	S	S	S
x21302-270	R	H	S	R	R	S	S	S
ISM	R	–	–	–	–	–	–	–
RG 170	–	–	–	–	–	–	–	–
TKM 13	S	S	S	S	S	S	S	S
AD (Bio) 09518	–	R	R	R	–	–	–	–
AD (Bio) 13066	–	R	R	R	–	–	–	–
CO 52	–	–	–	–	–	–	–	–
PTB 33	–	–	–	–	R	R	R	R

^*^R Resistance, *S* Susceptible, *H* Heterozygous

#### Growth and yield performance of selected resistant lines of the F_5_ population

The mean performance of the selected 51 resistant lines of the F_5_ population and the parental lines for the traits, days to 50% flowering, plant height, panicle length, number of productive tillers, single plant yield and thousand grain weight are listed in the supplementary Table 3.

In comparison to the parental lines, among the selected F_5_ line, x21302-241 which confers a high level of resistance reaction for BL and BB diseases was found with maximum PL, NPT, and SPY of 33.2 cm, 30 productive tillers, and 59.99 g respectively. The selected lines, x21302-239 which confers increased PL (30.8 cm) and also SPY over 4 parents. The line, x21301-52 which showed a promising resistant reaction was found with increased PH of 126.4 cm, and PL of 30.04 cm than the parents, and whereas the NPT was found to be reduced to 10 productive tillers, and SPY (27.44 g) was coincides with the average yield of parental lines. Out of 51 lines, x21301-150, x21302-241, x21302-243, and x21302-250 were identified with PL of more than 30 cm. The NPT of x21301-84, x21301-98, x21301-131, x21301-150, x21302-241, x21302-250, x21302-254 were recorded with more than 25 productive tillers. Two lines, x21302-240 and x21302-241 which found to have high level of resistance showed decreased (24.40 g) and increased (89.59 g) SPY over the parental lines respectively. Among the 51 F_5_ lines, nine lines, x21301-32, x21301-52, x21301-74, x21301-100, x21301-150, x21302-152, x21302-161 and x21302-239 were identified as agronomically superior lines with combined resistant reactions against multiple biotic stresses.

#### Estimation of variability parameter for F_5_ generation

The analysis of variation (ANOVA) among the F_5_ lines and checks showed significant differences for all the studied traits, namely days to 50% flowering (DFF), plant height (PH), panicle length (PL), number of productive tillers (NPT), single plant yield (SPY), 1000-grain weight (TGW) (Table 3).

**Table 3:** ANOVA for biometric and resistance traits of the F_5_ population shows the significant variation in the treatment

Source	df	Mean. Sq					
		DFF	PH	PL	NPT	SPY	TGW
Treatment (ignoring Blocks)	57	287.16**	585.59**	11.47**	47.76**	142.46**	16.83**
Treatment: Check	6	536.26**	829.78**	58.70**	183.05**	301.23**	78.15**
Treatment: Test	50	146.62**	305.26**	4.34*	32.47**	113.47**	6.94**
Treatment: Test vs. Check	1	5819.51**	13,137.21**	84.13**	0.75^ ns^	639.71**	143.04**
Block (eliminating Treatments)	4	0.74^ ns^	1.53^ ns^	1.33^ ns^	0.43^ ns^	10.44^ ns^	0.50^ ns^
Residuals	24	2.29	18.66	1.86	2.76	28.44	0.37

ⁿˢ*P * > 0.05; *P  <  = 0.05; **P  <  = 0.01

*df* Degrees of freedom, *DFF* Days to 50% flowering, *PH* Plant Height, *PL* Panicle Length, *NPT* Number of Productive Tillers, *SPY* Single Plant Yield, *TGW* 1000 grain weight

The GCV for the traits, DFF, PH, NPT, and SPY, was high. Whereas low and moderate GCV was observed for the traits PL and TGW respectively. The heritability estimates were high for DFF, PH, NPT, SPY, and TGW. Genetic advance as per cent of mean (GAM), was also high for the traits viz., DFF, PH, NPT, SPY, and TGW traits and low for PL (Table 4).

**Table 4:** Genetic variability parameters of the observed traits of F_5_ population

Trait	Mean	CV	PV	GV	EV	GCV	GCV category	PCV	PCV category	ECV	hBS	hBS category	GA	GAM	GAM category
DFF	109.04	1.45	146.62	144.32	2.29	11.02	Medium	11.10	Medium	1.39	98.44	High	24.59	22.55	High
PH	126.66	3.62	305.26	286.60	18.66	13.37	Medium	13.79	Medium	3.41	93.89	High	33.84	26.72	High
PL	27.14	5.14	4.34	2.48	1.86	5.81	Low	7.68	Low	5.03	57.14	Medium	2.46	9.05	Low
NPT	18.31	9.10	32.47	29.70	2.76	29.76	High	31.11	High	9.08	91.49	High	10.75	58.73	High
SPY	32.36	17.33	113.47	85.02	28.44	28.50	High	32.92	High	16.48	74.93	High	16.47	50.89	High
TGW	19.08	3.31	6.94	6.57	0.37	13.44	Medium	13.81	Medium	3.18	94.69	High	5.15	26.98	High

*CV* Coefficient of Variation, *PV* Phenotypic Variance, *GV* Genotypic Variance, *EV* Environmental Variance, *GCV* Genotypic Coefficient of Variation, *PCV* Phenotypic Coefficient of Variation, *ECV* Environmental Coefficient of Variation, *hBS* Heritability in Broad Sense, *GA* Genetic Advance, *GAM* Genetic Advance in percent of Mean, *DFF* Days to 50% flowering, *PH* Plant Height, *PL* Panicle Length, *NPT* Number of Productive Tillers, *SPY* Single Plant Yield, *TGW* 1000 grain weight

#### Estimation of correlation parameters (F_5_ Population)

**Fig. 7:**
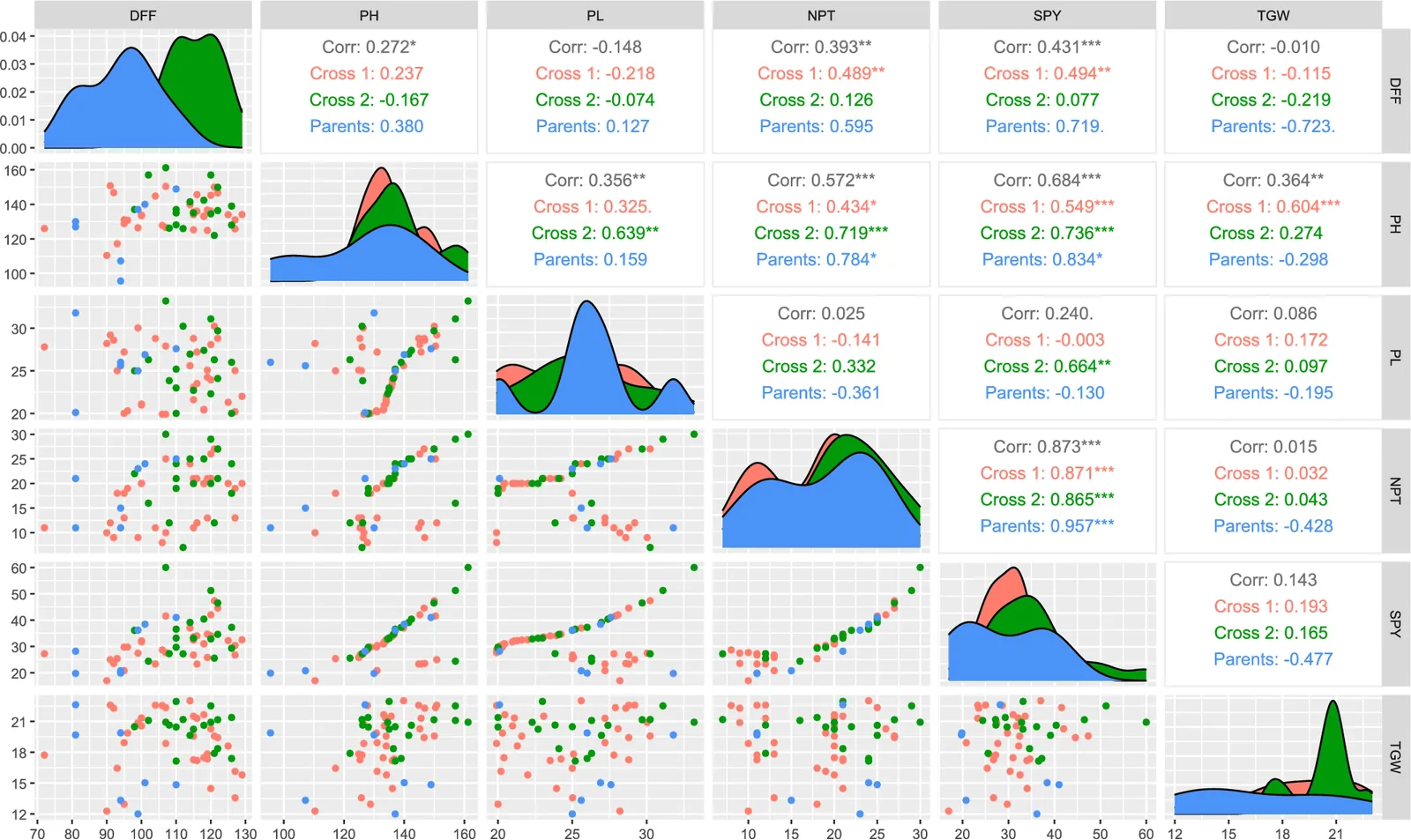
Correlation among the observed growth and yield-related traits. *DFF* Days to 50% flowering; *PH* Plant height, *PL* Panicle length, *NPT* Number of productive tillers, *SPY* Single plant yield, *TGW* Thousand grain weight (test weight)

Correlation studies among the F_5_ lines revealed a positive association between single plant yield (SPY) and had a significantly positive association with traits viz., DFF, PH, and NPT. The trait, PL was significantly correlated with the PH and PH had a significantly positive correlation with DFF, PL, NPT, SPY, and TGW. The NPT trait had a positive correlation with DFF (r = 0.393). while the remaining trait combinations recorded no correlation (Fig. 7).

#### Estimation of and principal component 

**Fig. 8:**
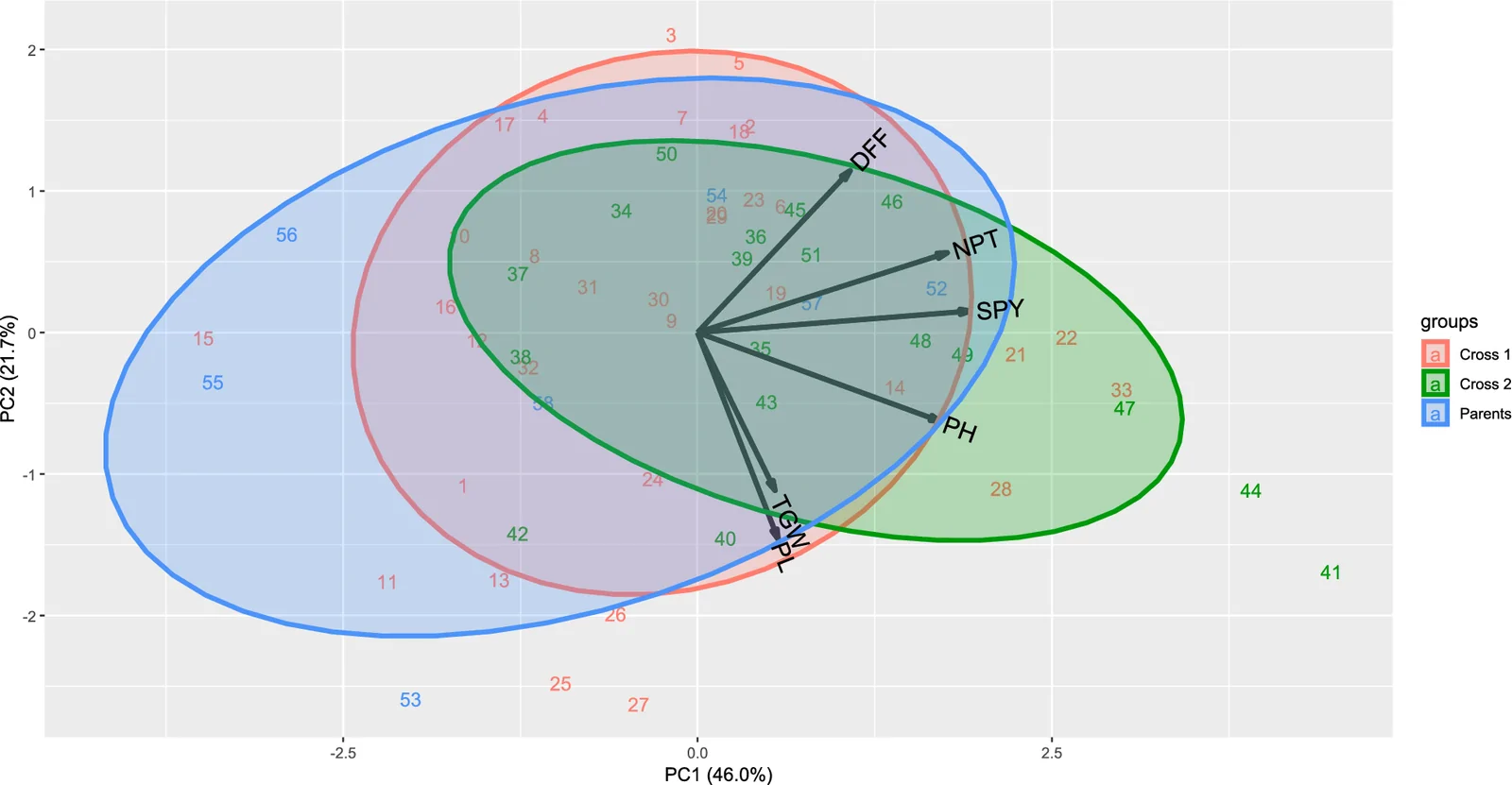
PCA biplot between PC1 and PC2 with variables. *PC* Principal components, *DFF* Days to 50% flowering, *PH* Plant height, *PL* Panicle length, *NPT* Number of productive tillers, *SPY* Single plant yield, *TGW* Thousand grain weight (test weight)

**Fig. 9:**
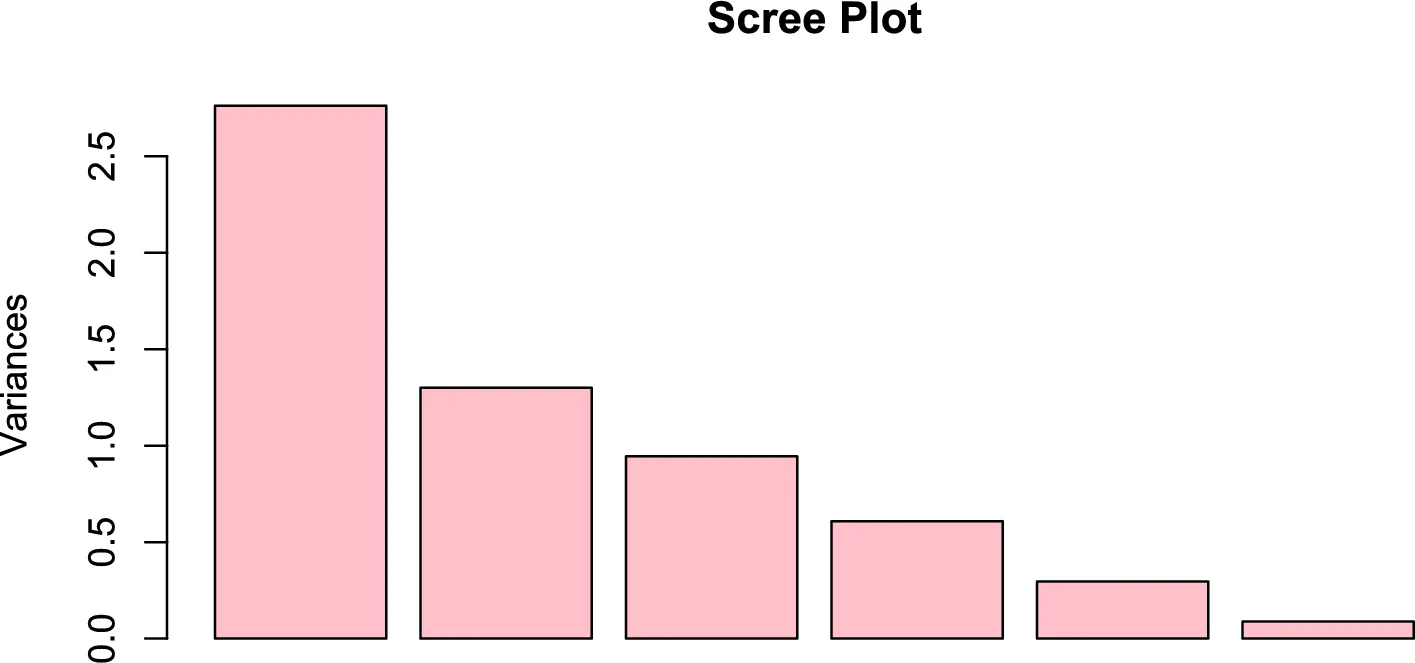
Scree plot between the variance and PC based on the eigenvalues of F_5_ population

A total of six principal components (PCs) were calculated from the F_5_ lines based on biometric traits. First, four PCs contributed 93.6% of the variance, each with an eigenvalue of more than 1. For the PC1, the traits with the largest loading of SPY were followed by NPT, PH, DFF, PL, and TGW. The second PC contributes 21.7% of variance to the total variance. The trait with the largest contribution for PC2 was panicle length, 1000-grain weight, and plant height. The PC3 and PC4 contributed 10.3% and 10.2% of variance to the total variance respectively. The traits, PL, NPT, and SPY contribute to PC3. The trait with the largest contribution was NPT followed by NPT (Fig. 8). Upon analyzing the vector plot, the single plant yield (SPY) was positively correlated with the DFF, NPT, PL, PH, and TGW with an acute angle of inclination. The trait DFF showed a negative correlation with TGW and PL, positioned in an obtuse angle (Fig. 9).

#### Estimation of genetic effect of resistance

Effect size analysis revealed substantial genotypic influence on disease resistance. Eta squared values were high for BL (η^2^ = 0.38), and BB (η^2^ = 0.39), indicating strong genetic control of resistance. In contrast, BPH resistance showed comparatively lower effect size (η^2^ = 0.14), suggesting a moderate genotypic contribution.

## Discussion

As the environmental conditions change over time, the dynamics of pathogen species and pathotypes, together with the populations of insects within ecosystems, are concurrently altered. Expansion of pathogen niches and insect populations in response to the rising temperature reduces paddy production and causes huge food insecurities in the future along with a significant impact on the economy of the country [51]. Changes in the precipitation level, rainfall pattern, and even severe drought or flood can create favorable environmental conditions for widespread disease epidemics [31]. The disease-causing pathogens spread and invade new territories as a result of climate change [24]. Therefore, the present study was carried out to develop multiple biotic stress resistant cultivar by introgression of resistant genes against blast, bacterial leaf blight and brown plant hopper through multi-parental crosses and marker-assisted selection.

Plants have developed several lines of defense mechanisms against biotic stresses caused by pathogens and insects. Upon the infection or infestation of a pathogen or insect, plants respond by physical barriers such as the deposition of cellulose in the cell wall, the thickening of the cuticular layer, etc. As a second line of resistance in the host plant, it is triggered by the pathogen called pathogen-associated molecular patterns (PAMPs) [41]. This defense mechanism is based on the discernment of the pathogenic structures by pathogen recognition receptors (PPRs) [65]. The resistance ‘R’ protein continuously monitors the cellular machinery and serves as guarding key virulence targets inside the cell. The interaction between the Avr products and the non-R cellular factors of the host was explained as the guard hypothesis. The R proteins discern the modified status of the target and induce rapid defense response (Marathe and Dinesh Kumar). Although these two lines of defense restrict the pathogen growth, the evolution of the pathogen improves the virulence and its related components. The pathogen injects effector proteins into the host plant cell that suppress the PAMP-triggered immunity (PTI) so that the pathogen can survive and complete its lifecycle. In the process of resistance, plants have developed a third level of defense called host resistance genes (*R* genes) which are activated by the pathogen effectors [62]. The improvement of resistance in the host plant by R genes is also favored by the decoy model of effector activities followed by these resistant genes. As the R genes are polymorphic i.e. irrespective of presence or absence in the different individuals of the population, natural selection favors the target guard effector with improved interaction to enhance the pathogen perception [56]. The products produced during the activation of resistance ‘R’ genes under the stress condition were classified into five groups, viz., detoxifying enzymes, kinases, NBS/LRR proteins, extracellular receptors, and receptor kinases [45]. So the development of rice varieties with resistant genes and QTLs is more important to tolerate or resist the evolving pathotypes and biotypes [63]. The main cause for the development of resistant varieties with superior agronomic traits is the ‘growth-defense trade-off’ [14]. Harboring two or more resistant genes for a specific disease or insect improves the durability and level of resistance in the host plant [64]. Development of multiple resistant lines against blast, bacterial leaf blight, false smut, and brown plant hopper [39, 43, 49, 58] through the marker-assisted gene pyramiding approach has been successfully performed in a single genotype with multiple resistance along with enhanced yield and grain quality traits [13, 17].

From the two different gene pyramided populations, four lines, x21301-38, x21302-151, x21302-240, and x21302-241 were identified as highly resistance to blast disease. The presence of the dominant blast-resistant gene, *Pi54* was validated through the gene-specific marker, Pi54-MAS. The activation of the major blast resistance gene *Pi54* activates 1154 genes, conferring resistance against the *Magnaphorthae oryzae* pathogen. The resistance mechanism involves increased activity of stress-responsive enzymes such as callose, laccase, phenylalanine ammonium lyase, peroxidase, lipoxygenase, chitinase, etc*.* [20]. The relative expression analysis of *Pi54* with an orthologue gene *Pi54of*, resulted in an increase of 23 folds at 72 hpi (hours post-inoculation) [15]. The Pi54 resistance gene favors the subcellular localization in the epidermal layers by up-regulation of callose synthesis genes. The callose content deposition in the epidermal layers blocks the plasmodesmata and helps in the blockage of fungal penetration into the plant cells through a papillae formation [4]. The defense enzyme phenylalanine ammonium lyase plays a crucial role in the biosynthetic pathway of phytoalexins and lignin, preventing the fungal pathogen’s cell wall penetration. In the current study, biochemical analysis in the blast resistant lines showed increased levels of defense enzyme such as phenylalanine ammonium lyase (PAL), peroxidase (POX), and ascorbate peroxidase (APX). Significantly highest PAL enzymatic activity was recorded in x21302-241 (2.071 folds) followed by x21301-52 (1.989 folds), x21301-38 (1.960 folds), and x2102-240 (1.950 folds). PAL activity showed an average of 1.953 folds increase in the selected blast-resistant lines in comparison with the disease free controlled condition at 15 DAI, the level of increase is close to the resistant check, ISM in which 2.05 folds increase was observed. The activity of POX and APX among the test lines in the pathogen-inoculated condition was increased by an average of 6.57 folds and 2.16 folds respectively. The peroxidase enzyme was found to be upregulated with the activation of the Pi54 gene, which is involved in the biosynthetic pathway of lignin, lignification, and pathogen defense [20]. For the growth and development of the pathogen, it reduces the host sugar levels which results in the induction of sugar-cleaving enzymes like sucrose synthase, invertase, and amylases [34]. It also increases the regulation of signal-transducing phytohormones such as salicylic acid, jasmonic acid, and ethylene [16, 20].

Total of 11 bacterial leaf blight resistance genes namely, *Xa1*, *Xa3*, *Xa4*, *xa5*, *Xa10*, *xa13*, *Xa21*, *Xa23*, *xa25*, *Xa27*, and *xa41* were cloned [28] and classified into four groups based on their encoding property, including receptor-like kinase genes, sugar will eventually be exported transporter (*SWEET*) genes, executor genes, and other types of genes [54]. The dominant resistant gene, *Xa21* confers a broad spectrum of resistance against the *Xoo* pathogen. The recessive *R* genes, *xa13*, *xa25*, and *xa41* develop improved resistance in combination with other dominant resistant genes. These genes encode for the clade III SWEET proteins. From the BB resistance screening, 10 highly resistant lines were identified, genotyping reveals that these lines carry different combinations of resistant genes viz., *xa5*, *xa13*, and *Xa21*. The level of resistance conferred by the *Xa21* resistance gene increases from the two-leaf stage and attains a higher level of resistance reaction at the adult plant stage [53, 57]. Under the pathogen infection condition, the cellular signaling processes in plants are mainly mediated by the serine-threonine kinase or by the leucine-rich repeats domain, suggesting a role in protein phosphorylation or protein–protein interaction. The *Xa21* resistant gene was revealed with 1025 amino acid sequences with variable regions and domains such as the LRR region, intracellular protein kinase catalytic domain, etc. The sub-domains VI and VII, which are conserved amino acid sequences of DLKPEN and G(T/S)XX(Y/F)XAPE, indicate serine-threonine specificity [53]. The combination of *xa5*, *xa13,* and *Xa21* genes was found to exhibit a high level of resistance reaction in the x21302-241 line. Likewise, the *xa5* showed higher level resistance to the *Xoo* pathogen in combination with *Xa21* in the line, x21302-240.

The host plant resistance genes to the brown plant hopper insect were mapped on several chromosomal regions in clusters, with 25 BPH-resistant genes mainly distributed on chromosomes 3, 4, 6, and 12. As the host responds to the infection of the pathogen, PTI and ETI resistance mechanisms are triggered by the pathogen’s infestation. While under the BPH infestation, herbivore-associated molecular patterns (HAMP) or damage-associated molecular patterns (DAMP) were also triggered as an initial phase of the resistance mechanism [30]. PTB 33 is the most common resistant donor in the BPH resistance breeding program in India, which confers resistance against biotype 4. Upon the introgression of BPH resistance genes, such as *Bph1*, *bph2*, and *Bph17* six lines were identified as resistant and moderately resistant lines. The hormonal signaling pathway is effected by jasmonic acid, ethylene, and salicylic acid, which regulate the immune response of the host. As a primary structural defense response, the HAMP triggers the callose deposition of the sieve tube, synthesis of proteinase inhibitor, and production of secondary metabolites, which induce the antixenosis and antibiosis effects on BPH [9]. The BPH resistance genes show a high level of antibiosis and antixenosis resistance mechanisms [38]. The honeydew area test reveals the feeding activity of the adult insects upon the selected resistant and moderately resistant lines. The lines, x21302-239, x21301-52, and x21302-270 showed the lowest honeydew deposition area compared to the resistant check, PTB 33. The feeding activity of the BPH insect varies with each cultivar based on their level of resistance. The insect spends more time in the susceptible cultivar whereas in the resistant cultivar, it spends a very short time [60]. From the study, line x21302-239 showed a resistant reaction with reduced honeydew area along with a low nymphal preference rate. Most of the identified resistant lines showed superior agronomic performance with required single plant yield. The line, x21301-239, which confers resistance to blast, BB, and BPH, also showed reasonable agronomic performance with a single plant yield of 33.15 g. Precise selection of lines carrying multiple resistance genes in this study was majorly attributed through the deployment of marker-assisted selection. Among the targeted resistance genes, *Pi54*, *xa5*, *xa13*, *Bph1*, *bph2*, and *Bph17* were favorably validated with the molecular markers. Besides confirmation, molecular markers can distinguish the heterozygous and homozygous state of targeted genes. Identification of plants carrying homozygous resistance genes in advanced generations uplifts the positive selection process, which shifts the population towards the homogenous state. On the other hand, MAS also favored in selection of individuals carrying recessive resistance genes, which is usually a difficult process through conventional approaches. Since, the level of reproduction of segregants with recessive resistance genes is not the same as that of dominant genes [11]. The combination of different genes confers resistance at variable intensities. The observed variation in resistance levels is influenced by the genetic background and the specific combinations of genes involved, likely due to masking or interaction effects among resistance loci. The MAS provides substantial support for the improved or durable resistance of a particular effective combination of resistance genes [5]. For instance in this study, the resistance and moderate resistance reaction found in the line x21302-239, supported the horizontal resistance to BL, BB and BPH due to the presence of *Pi54*, *Xa21*, *xa5*, *xa13*, *Bph1*, *bph2*, and *Bph17genes*. This study highlighted the potentiality of the powerful breeding tools such as multi – parental crossing and marker-assisted selection combined with precise phenotyping to develop multiple biotic stress-tolerant rice varieties.

## Conclusion

The present study resulted in the identification of breeding lines with multiple resistance genes namely, *Pi54*, *xa5*, *xa13*, *Xa21*, *Bph1*, *bph2*, *Bph3*, and *Bph17* against BL, BB, and BPH. Through multi-parental hybridization and early-generation MAS, precise pyramiding and tracking of these genes were achieved from the F_1_ generation onward, enabling the development of advanced lines with stacked resistance loci. A principal innovation of this work is the successful pyramiding of up to seven resistance genes within single breeding lines, coupled with validation of their agronomic compatibility. Promising line, x21302-239 can be used as a donor parent for the BL, BB, and BPH resistance breeding programme. Breeding line x21302-239 is not suitable for the development of slender grain–type varieties, as it possesses short, bold grains with hard gel consistency (gel consistency = 10). However, owing to its intermediate amylose content (21.06%), it is well suited for the development of bold grain–type varieties. Along with a high level of resistance reaction against BL, BB, and BPH, the desirable agronomic traits and grain type of the lines, such as x21301-38, x21302-151, x21302-240, and x21302-241 favor generation advancement for varietal development. Since the virulence of the pathotype/biotype is bound with the coexistence of alleles of resistance genes and the host’s genetic background, the homozygous genetic background of the host causes an epidemic outbreak in a short span. In that event, multiple genetic backgrounds over a single population cause a negative impact on the pathogen’s infectious cycle, so that the epidemic development period can be reduced. Under this condition, several lines derived from the multiple parental lines that are recognized for their resistance and exhibit distinctive assortments of resistance genes serve as pre-breeding lines for future resistance breeding programmes in rice. Future research should prioritize multi-environment validation trials to assess long-term resistance durability and yield performance. Optimization of gene combinations to minimize adverse agronomic trade-offs, particularly those affecting plant morphology and grain quality, will be essential. In addition, functional genomic analyses investigating regulatory interactions and expression dynamics among pyramided resistance genes will provide mechanistic insights to guide the rational design of durable, multi-resistant rice cultivars.

## Supplementary Information

Supplementary Material 1 (DOCX 2432 KB)

Supplementary Material 2 (DOCX 35 KB)

## Data Availability

All data analyzed during this study are included in this manuscript file and its supplementary information file.

## Abbreviations

**APX**: Ascorbate peroxidase
**BL**: Blast
**BB**: Bacterial leaf blight
**BPH**: Brown plant hopper
**CTAB**: Cetyltrimethylammonium bromide
**DAMP**: Damage-associated molecular pattern
**ETI**: Effector triggered immunity
**HAMP**: Hormone-associated molecular patterns
**MAS**: Marker-assisted selection
**NBS/LRR**: Nucleotide binding site/Leucine Rich Repeats
**PAL**: Phenylalanine ammonium lyase
**PAMP**: Pathogen-associated molecular pattern
**POX**: Peroxidase
**PRR**: Pathogen recognition receptors
**PTI**: Pattern triggered immunity
**QTL**: Quantitative trait loci
**SWEET**: Sugar will eventually be exported transporter
