# Supplementary material for: A successful stacking of multiple biotic stress resistance in rice facilitated by genes and precision breeding tools
Source: Biol Res. 2026 Aug 5;59:48. doi: 10.1186/s40659-026-00710-0 (PMC13491621; doi:10.1186/s40659-026-00710-0)
Supplement: Supplementary file 1 [file 40659_2026_710_MOESM1_ESM.docx]

**Supplementary Fig. 1:** Molecular validation of resistance genes (*xa13* and *Xa21*) with the gene-specific molecular markers
