# Supplementary material for: A successful stacking of multiple biotic stress resistance in rice facilitated by genes and precision breeding tools
Source: Biol Res. 2026 Aug 5;59:48. doi: 10.1186/s40659-026-00710-0 (PMC13491621; doi:10.1186/s40659-026-00710-0)
Supplement: Supplementary file 2 [file 40659_2026_710_MOESM2_ESM.docx]

Table S1: Characteristics of parental lines used in the breeding program

| **S.No.** | **Genotypes** | **Source** | **Salient features** | **Reference** |
| --- | --- | --- | --- | --- |
| 1, | APD 19026: Improved Samba Mahsuri (ISM) | IIRR, Hyderabad | Medium slender genotype with blast resistance gene *Pi54* | (Rekha *et al.*, 2018) |
| 2. | RG 170 | TNAU, Coimbatore | Germplasm line having QTLs resistance to false smut | (Ramyaselvi *et al.*, 2023) |
| 3. | TKM 13 | Rice Research Station, Tirur, | Medium slender variety with good milling and cooking properties | (Banumathy *et al.*, 2016) |
| 4. | AD (Bio) 09518 | TNAU, Coimbatore | Advanced breeding lines having genes *viz*., *xa5*, *xa13* and *Xa21* for BLB resistance | (Sakthivel *et al.*, 2017) |
| 5. | AD (Bio) 13066 | TNAU, Coimbatore | Advanced breeding lines having genes *viz*., *xa5*, *xa13* and *Xa21* for BLB resistance | (Sakthivel *et al.*, 2017) |
| 6. | CO 52 | TNAU, Coimbatore | Medium slender variety with good milling and cooking properties | (Robin *et al.*, 2019) |
| 7 | PTB 33 | RRS, Patambi, Kerala | Photo-sensitive, red-seeded variety with genes *Bph3* and *Bph17* for BPH resistance | (Cheng *et al.*, 2021) |

Table S2: Percent disease incidence of resistance score for BL, BB and BPH stresses

| **Lines** | **Percent disease incidence (%)** | | |  | **Lines** | | **Percent disease incidence (%)** | | |
| --- | --- | --- | --- | --- | --- | --- | --- | --- | --- |
|  | **BL** | **BB** | **BPH** |  |  |  | **BL** | **BB** | **BPH** |
| x21301-1 | 24.44 | 24.44 | 66.67 |  | x21301-146 | | 77.78 | 28.89 | 77.78 |
| x21301-2 | 42.22 | 42.22 | 77.78 |  | x21301-147 | | 51.11 | 37.78 | 33.33 |
| x21301-5 | 37.78 | 37.78 | 88.89 |  | x21301-150 | | 24.44 | 46.67 | 33.33 |
| x21301-20 | 20.00 | 20.00 | 66.67 |  | x21302-151 | | 24.44 | 55.56 | 66.67 |
| x21301-26 | 24.44 | 24.44 | 55.56 |  | x21302-152 | | 11.11 | 64.44 | 88.89 |
| x21301-32 | 11.11 | 24.44 | 100.00 |  | x21302-153 | | 33.33 | 64.44 | 66.67 |
| x21301-33 | 28.89 | 24.44 | 77.78 |  | x21302-161 | | 11.11 | 55.56 | 66.67 |
| x21301-38 | 20.00 | 24.44 | 88.89 |  | x21302-164 | | 37.78 | 5.56 | 66.67 |
| x21301-41 | 28.89 | 24.44 | 66.67 |  | x21302-239 | | 20.00 | 46.67 | 33.33 |
| x21301-44 | 24.44 | 11.11 | 100.00 |  | x21302-240 | | 11.11 | 33.33 | 100.00 |
| x21301-47 | 64.44 | 11.11 | 88.89 |  | x21302-241 | | 11.11 | 28.89 | 100.00 |
| x21301-51 | 33.33 | 11.11 | 88.89 |  | x21302-243 | | 20.00 | 28.89 | 77.78 |
| x21301-52 | 11.11 | 11.11 | 33.33 |  | x21302-246 | | 28.89 | 20.00 | 66.67 |
| x21301-53 | 37.78 | 11.11 | 66.67 |  | x21302-250 | | 33.33 | 15.56 | 66.67 |
| x21301-57 | 37.78 | 28.89 | 77.78 |  | x21302-251 | | 46.67 | 11.11 | 88.89 |
| x21301-59 | 55.56 | 28.89 | 100.00 |  | x21302-252 | | 33.33 | 11.11 | 33.33 |
| x21301-64 | 28.89 | 28.89 | 100.00 |  | x21302-254 | | 37.78 | 11.11 | 88.89 |
| x21301-73 | 20.00 | 28.89 | 77.78 |  | x21302-264 | | 37.78 | 15.56 | 88.89 |
| x21301-74 | 11.11 | 28.89 | 100.00 |  | x21302-266 | | 24.44 | 20.00 | 77.78 |
| x21301-83 | 28.89 | 20.00 | 88.89 |  | x21302-268 | | 28.89 | 37.78 | 100.00 |
| x21301-84 | 20.00 | 20.00 | 66.67 |  | x21302-270 | | 55.56 | 37.78 | 33.33 |
| x21301-98 | 15.56 | 20.00 | 77.78 |  | APD 19026 | | 11.11 | 11.11 | 100.00 |
| x21301-99 | 51.11 | 20.00 | 100.00 |  | RG170 | | 11.11 | 11.11 | 100.00 |
| x21301-100 | 15.56 | 24.44 | 100.00 |  | TKM13 | | 73.33 | 73.33 | 100.00 |
| x21301-107 | 28.89 | 28.89 | 77.78 |  | AD (Bio) 09518 | | 11.11 | 11.11 | 100.00 |
| x21301-112 | 28.89 | 28.89 | 77.78 |  | AD (Bio) 13064 | | 11.11 | 11.11 | 100.00 |
| x21301-116 | 37.78 | 28.89 | 77.78 |  | CO 52 | | 11.11 | 11.11 | 100.00 |
| x21301-131 | 28.89 | 28.89 | 100.00 |  | PTB 33 | | 64.44 | 64.44 | 11.11 |
| x21301-132 | 20.00 | 28.89 | 88.89 |  | TN 1 | | - | - | 100.00 |
| x21301-133 | 24.44 | 24.44 | 77.78 |  | |  |  |  |  |

BL – Blast; BB – Bacterial Leaf Blight; BPH – Brown Plant Hopper

Table S3: Confirmatory screening of identified resistant lines for BL, BB and BPH stresses

| **Lines** | **Percent disease incidence (%)** | | |  | **Lines** | | **Percent disease incidence (%)** | | | | | |  |
| --- | --- | --- | --- | --- | --- | --- | --- | --- | --- | --- | --- | --- | --- |
|  | **BL** | **BB** | **BPH** |  |  |  | **BL** | | **BB** | | **BPH** | |  |
| x21301-1 | 33.33 | 46.67 | 77.78 |  | x21301-146 | | 28.89 | | 24.44 | | 88.89 | |  |
| x21301-2 | 22.22 | 37.78 | 88.89 |  | x21301-147 | | 33.33 | | 11.11 | | 33.33 | |  |
| x21301-5 | 33.33 | 33.33 | 100.00 |  | x21301-150 | | 22.22 | | 11.11 | | 33.33 | |  |
| x21301-20 | 28.89 | 37.78 | 77.78 |  | x21302-151 | | 11.11 | | 11.11 | | 77.78 | |  |
| x21301-26 | 24.44 | 11.11 | 66.67 |  | x21302-152 | | 28.89 | | 11.11 | | 100.00 | |  |
| x21301-32 | 33.33 | 24.44 | 100.00 |  | x21302-153 | | 33.33 | | 51.11 | | 77.78 | |  |
| x21301-33 | 33.33 | 24.44 | 88.89 |  | x21302-161 | | 33.33 | | 11.11 | | 66.67 | |  |
| x21301-38 | 11.11 | 11.11 | 100.00 |  | x21302-164 | | 31.11 | | 64.44 | | 88.89 | |  |
| x21301-41 | 22.22 | 11.11 | 55.56 |  | x21302-239 | | 20.00 | | 33.33 | | 33.33 | |  |
| x21301-44 | 33.33 | 11.11 | 100.00 |  | x21302-240 | | 11.11 | | 11.11 | | 100.00 | |  |
| x21301-47 | 28.89 | 11.11 | 77.78 |  | x21302-241 | | 11.11 | | 11.11 | | 100.00 | |  |
| x21301-51 | 33.33 | 11.11 | 55.56 |  | x21302-243 | | 33.33 | | 24.44 | | 33.33 | |  |
| x21301-52 | 20.00 | 11.11 | 33.33 |  | x21302-246 | | 22.22 | | 51.11 | | 33.33 | |  |
| x21301-53 | 22.22 | 11.11 | 33.33 |  | x21302-250 | | 33.33 | | 46.67 | | 66.67 | |  |
| x21301-57 | 28.89 | 77.78 | 100.00 |  | x21302-251 | | 31.11 | | 33.33 | | 100.00 | |  |
| x21301-59 | 33.33 | 24.44 | 100.00 |  | x21302-252 | | 33.33 | | 28.89 | | 55.56 | |  |
| x21301-64 | 28.89 | 24.44 | 100.00 |  | x21302-254 | | 33.33 | | 86.67 | | 88.89 | |  |
| x21301-73 | 33.33 | 24.44 | 100.00 |  | x21302-264 | | 28.89 | | 60.00 | | 33.33 | |  |
| x21301-74 | 33.33 | 11.11 | 100.00 |  | x21302-266 | | 33.33 | | 46.67 | | 77.78 | |  |
| x21301-83 | 33.33 | 77.78 | 100.00 |  | x21302-268 | | 33.33 | | 11.11 | | 100.00 | |  |
| x21301-84 | 28.89 | 11.11 | 77.78 |  | x21302-270 | | 31.11 | | 24.44 | | 55.56 | |  |
| x21301-98 | 33.33 | 24.44 | 88.89 |  | APD 19026 | | 11.11 | | 11.11 | | 100.00 | |  |
| x21301-99 | 22.22 | 11.11 | 100.00 |  | RG170 | | 55.56 | | 11.11 | | 100.00 | |  |
| x21301-100 | 33.33 | 15.56 | 100.00 |  | TKM13 | | 77.78 | | 77.78 | | 100.00 | |  |
| x21301-107 | 31.11 | 33.33 | 100.00 |  | AD (Bio) 09518 | | 55.56 | | 11.11 | | 100.00 | |  |
| x21301-112 | 33.33 | 46.67 | 22.22 |  | AD (Bio) 13064 | | 55.56 | | 11.11 | | 100.00 | |  |
| x21301-116 | 33.33 | 15.56 | 100.00 |  | CO 52 | | 11.11 | | 11.11 | | 100.00 | |  |
| x21301-131 | 20.00 | 51.11 | 100.00 |  | PTB 33 | | 77.78 | | 68.89 | | 11.11 | |  |
| x21301-132 | 22.22 | 15.56 | 33.33 |  | TN 1 | | - | | - | | 100.00 | |  |
| x21301-133 | 33.33 | 51.11 | 77.78 |  | |  | |  | |  | |  | |

BL – Blast; BB – Bacterial Leaf Blight; BPH – Brown Plant Hopper
